# Acute Bacterial Meningitis in Qatar: A Hospital-Based Study from 2009 to 2013

**DOI:** 10.1155/2017/2975610

**Published:** 2017-07-13

**Authors:** Fahmi Yousef Khan, Mohammed Abu-Khattab, Eman Abdulrahman Almaslamani, Abubaker Ahmed Hassan, Shehab Fareed Mohamed, Abdurrahman Ali Elbuzdi, Nada Yagoub Elmaki, Deshmukh Anand, Doiphode Sanjay

**Affiliations:** ^1^Department of Medicine, Hamad General Hospital, Doha, Qatar; ^2^Department of Medicine, Infectious Disease Division, Hamad General Hospital, Doha, Qatar; ^3^Department of Pediatrics, Infectious Disease Division, Hamad Medical Corporation, Sedra, Doha, Qatar; ^4^Hematology Division, Al Amal Hospital, Doha, Qatar; ^5^Medical Intensive Care Unit, Hamad General Hospital, Doha, Qatar; ^6^Department of Microbiology, Hamad General Hospital, Doha, Qatar

## Abstract

**Background and Objectives:**

Bacterial meningitis is a common medical condition in Qatar. The aim of this study was to describe the clinical characteristics of bacterial meningitis, the frequency of each pathogen, and its sensitivity to antibiotics and risk factors for death.

**Patients and Methods:**

This retrospective study was conducted at Hamad General Hospital between January 1, 2009, and December 31, 2013.

**Results:**

We identified 117 episodes of acute bacterial meningitis in 110 patients. Their mean age was 26.4 ± 22.3 years (range: 2–74) and 81 (69.2%) of them were male patients. Fifty-nine episodes (50.4%) were community-acquired infection and fever was the most frequent symptom (94%), whereas neurosurgery is the most common underlying condition. Coagulase-negative staphylococci were the most common causative agent, of which 95% were oxacillin-resistant, while 63.3% of* Acinetobacter* spp. showed resistance to meropenem. The in-hospital mortality was 14 (12%). Only the presence of underlying diseases, hypotension, and inappropriate treatment were found to be independent predictors of mortality.

**Conclusion:**

Acute bacterial meningitis predominantly affected adults and coagulase-negative staphylococci species were the common causative agent in Qatar with majority of infections occurring nosocomially. More than 90% of all implicated coagulase-negative staphylococci strains were oxacillin-resistant.

## 1. Introduction 

Despite medical advances, acute bacterial meningitis (ABM) constitutes a global public health problem, especially in developing countries with poor health facilities due to high rates of malnutrition, poor living conditions, and lack of access to appropriate preventive and curative services that may predispose people to the disease and reduce their chances of receiving optimal treatment [1, 2]. In developed countries, the burden of the disease has reduced and its epidemiology has changed as a result of the widespread use of vaccines against the most common meningeal pathogens [3].

Accurate information on important etiologic agents and populations at risk is needed to determine public health measures and ensure appropriate management of ABM [3]. In Qatar, although ABM is a common medical condition that physicians face, there are few reports describing this disease [4–6]. We conducted the present study, the purposes of which were to (1) describe the demographic and clinical characteristics of ABM, (2) determine the relative frequency of each pathogen and its susceptibility to various antimicrobial agents, and (3) determine the outcome and the significant predictors of the outcome among patients with ABM in Qatar.

## 2. Materials and Methods

### 2.1. Design and Setting

This retrospective descriptive study, which involved all in-patients with ABM, was conducted at Hamad General Hospital between January 1, 2009, and December 31, 2013. This hospital is a 603-bed tertiary care center that covers all specialties except for hematology-oncology, cardiology, and obstetrics and it has been Joint Commission International (JCI) accredited since 2006 and is the first hospital system in the region to achieve institutional accreditation from the Accreditation Council for Graduate Medical Education-International (ACGME-I). Currently, there are three adult ICUs in Hamad General Hospital, namely, Medical ICU (MICU) with 22 beds, Surgical ICU (SICU) with 12 beds, and Trauma ICU (TICU) with 15 beds.

### 2.2. Definitions

ABM was diagnosed on the basis of at least one of the following compatible clinical pictures with no other apparent cause: fever (38°C), headache, meningeal signs, cranial nerve signs, and impaired mental status, plus one of the following [7, 8]:

1. Positive cerebrospinal fluid (CSF) culture
2. Positive CSF bacterial antigen test (with latex agglutination counterimmunoelectrophoresis) associated with pleocytosis mainly neutrophilic, defined as absolute WBC ≥ 100 cells/mm^3^, with a decreased glucose level ≤ 40 mg/dL and an increased protein concentration ≥ 60 mg/dL.

ABM was considered nosocomial if the diagnosis was made after more than 48 hours of hospitalization or within a short period of time (i.e., usually within one month after discharge from the hospital where the patient had received an invasive procedure, especially a neurosurgical procedure) [9]. On the other hand, ABM was considered as community-acquired if the diagnosis was made within the first 48 hours of hospitalization and the patient was not hospitalized in the preceding month [10]. Empirical antimicrobial therapy was deemed to be inappropriate if the antibiotics were administered more than 24 hours after CSF collection and/or when the dosage, route, and duration of treatment were not in accordance with hospital guidelines [11]. Hypotension was defined as blood pressure < 90/60 mmHg. Multidrug-resistant organisms are defined as microorganisms, predominantly bacteria, that are resistant to one or more classes of antimicrobial agents [12].

Viral, fungal, mycobacterial, polymicrobial, and drug induced meningitis were excluded. ABM episodes with the same organism were included only once. Coagulase-negative staphylococci and viridans streptococci are considered as causative agents if CSF showed pleocytosis mainly neutrophilic, defined as absolute WBC ≥ 100 cells/mm^3^, or a decreased glucose level ≤ 40 mg/dL or an increased protein concentration ≥ 60 mg/dL. The primary outcome was in-hospital mortality which included all causes of death during admission.

### 2.3. Isolation, Identification, and Antimicrobial Susceptibility Test of Microorganisms

Identification of isolates was based on colony morphology, Gram stain, oxidase, catalase, VITEK 2 Compact (bioMérieux, Durham, USA), and Phoenix (Becton Dickinson, NJ, USA). The antimicrobial minimal inhibitory concentrations (MICs) for the isolates were determined by using Phoenix (Becton Dickinson, NJ, USA) for GNB and staphylococci and enterococci (among Gram-positive cocci). For fastidious bacteria, susceptibility was determined with a gradient strip method (*E*-test strips, bioMérieux, Marcy-l'Étoile, France). The breakpoint interpretation was determined according to the recommendations of the Clinical Laboratory Standards Institute (CLSI) [13].

### 2.4. Source of Data and Data Collection

Cases were identified via hospital's discharge records, infection control records, and cerebrospinal fluid records maintained by the microbiology unit. These records were reviewed carefully by two investigators, in order not to miss any case. Records of all patients with bacterial meningitis were reviewed retrospectively to retrieve data on patients' demography, sign-symptoms, underlying medical conditions, investigations, names of microorganisms and their drug susceptibility, name and duration of therapy offered, appropriateness of therapy, and outcome.

### 2.5. Statistical Analysis

Quantitative variables were expressed as mean ± SD. Univariate logistic regression was performed to determine the probable predictors of in-hospital mortality. All potential risk factors at ≤0.1 level in the univariate analysis were entered in the multiple logistic regression to identify the independent predictors of mortality at *P* < 0.05. The data were analyzed with SPSS software (v 17; IBM Corp., Armonk, NY, USA).

### 2.6. Ethical Approval

Ethical approval (#13254/13) and a waiver of informed consent were obtained from the medical research ethical committee at Hamad Medical Corporation, Qatar.

## 3. Results

### 3.1. Demographic and Clinical Data

During the study period, we identified 117 episodes of ABM in 110 patients. There were 43, 22, 21, 12, and 18 episodes in 2009, 2010, 2011, 2012, and 2013, respectively. The study sample comprised 81 (69.2%) male and 29 (30.8%) female patients. Their mean age was 26.4 ± 22.3 years (range: 2–74), and 28 (23.9%) patients were Qatari. The peak frequency of ABM episodes was noted among adults (15–64 years old) (92.3%) (see Table 1). From a clinical point of view, fever was the most frequent symptom (110, 94%), followed by mental alteration (55, 47%), headache (43, 36.8%), and vomiting (35, 29.9%). Moreover, meningismus was detected in 31 (26.5%) patients (see Table 1).

### 3.2. Underlying Conditions

The most frequent underlying conditions were neurosurgery (54, 46.2%), hypertension (26, 22.2%), and diabetes mellitus (9, 7.7%) (see Table 1).

### 3.3. Cerebrospinal Fluid (CSF) Findings

The CSF findings of the 117 ABM episodes are listed in Table 2.

### 3.4. Setting of Infection and Types and Distributions of the Microorganisms

Fifty-nine episodes (50.4%) were community-acquired and the other 58 (49.6%) were nosocomially acquired ABM (see Table 3). The causative pathogens of the 117 enrolled ABM episodes are listed in Tables 3 and 4. Gram-positive pathogens accounted for 62 (53%) episodes and Gram-negative pathogens accounted for the other 55 (47%). In general, the most common causative agent of ABM in our cohort was coagulase-negative staphylococci; however, among the 59 community-acquired meningitis cases, the most common etiological agent was* Streptococcus pneumoniae*, whereas coagulase-negative staphylococci species were the leading cause of nosocomially acquired ABM. Among the implicated Gram-positive pathogens, coagulase-negative staphylococci were the most common (20, 17%), followed by* Streptococcus pneumoniae* (19, 16.2%). Among Gram-negative pathogens,* Klebsiella pneumoniae* was the most common (12, 10.2%) followed by* Neisseria meningitidis* (11, 9.4%).

### 3.5. Trends of Antimicrobial Susceptibility

Details of antimicrobial susceptibility are shown in Tables 5 and 6. Among the Gram-positive cases, 3 (18.6%) episodes of* Streptococcus pneumoniae* were resistant to ceftriaxone, while out of all coagulase-negative staphylococci isolates, 19 (95%) were methicillin-resistant. Among the Gram-negative cases, 100% of* Chryseobacterium* species were resistant to meropenem and colistin, while 63.3% of* Acinetobacter* species showed resistance to meropenem but none for colistin. All* Pseudomonas* spp. were sensitive to piperacillin-tazobactam and meropenem. Among* Klebsiella* isolates, 2 (16.6%) were extended spectrum beta-lactamase (ESBL) producers, but all of them were sensitive to meropenem and colistin. Of the 11 episodes with* Neisseria meningitides* infection, rifampicin resistance was found in 2 (25%). Multidrug resistance was observed in 38 (32.4%) of all episodes.

### 3.6. Treatment and Outcome

Antimicrobial treatment was initiated for all patients. Ceftriaxone plus vancomycin combination was the most widely used antimicrobial treatment followed by meropenem. Empiric therapy was inappropriate in 20 (17.1%) episodes. The crude in-hospital mortality in our study was 14 (12%).

### 3.7. Univariate and Multivariate Logistic Regression Analysis of Factors Associated with Death

By the univariate analysis, the following variables were found to be probable predictors of in-hospital mortality: presence of underlying diseases, nosocomial infection, multidrug-resistant episodes, hypotension, mental alteration, and inappropriate treatments (see Table 7). Only the presence of underlying diseases, hypotension, and inappropriate treatment were found to be independent predictors of mortality by multivariate logistic regression analysis (see Table 8).

## 4. Discussion

Acute bacterial meningitis is a serious disease which necessitates early diagnosis and aggressive therapy to improve prognosis. Regional information regarding demographic data of patients, associated underlying conditions, etiology, and antimicrobial susceptibility is essential for correct and timely management of this disorder. Our study was the first to attempt to determine the clinical picture and the spectrum of pathogens of bacterial meningitis in patients of all ages in Qatar.

This retrospective series revealed some observations that deserve attention: firstly, in contrast with the previous study [6], the trend was seen to decrease from 2009 to 2013. Among the total 117 episodes, 43 (36.7%) were reported in the year 2009, which decreased to 18 (15.4%) in 2013. Furthermore, the disease in our series predominantly affected adults rather than infants and young children. This picture is similar to what was found in west countries and it may be attributed to vaccine-related decline in* H*.* influenzae* and pneumococcal diseases [3, 9, 14, 15]. These data show that adults are the main target population which requires interventions to prevent and control diseases in Qatar.

Secondly, sex distribution of the disease showed male predominance in agreement with the previous report [6] and other reports from different countries [9, 10, 16–19]. The reason for this is obscure, and further studies are needed to identify the cause.

Thirdly, compared with the previous studies [4, 6], changes of common causative pathogens of ABM had been noted in our series. Coagulase-negative staphylococci species were the most common causative agents followed by* Streptococcus pneumoniae*. This can be explained by the expansion of neurosurgical services in our hospital with a consequent increase in the number of patients with postneurosurgical state. Similarly, reports from Taiwan [15–18] showed that there has been an increasing incidence of staphylococcal infection in ABM patients. However, in agreement with many reports worldwide [2, 7, 20–23],* Streptococcus pneumoniae *remain the common causative agent for community-acquired infection in our study.

Fourthly, drug resistance pattern showed that 95% of the implicated coagulase-negative staphylococci species were oxacillin-resistant and 63.3% of the implicated* Acinetobacter* species were meropenem-resistant. Both infections were predominantly nosocomial, which raised doubt regarding the infection control program in our hospital. Moreover, these findings result in therapeutic challenge in the choice of empiric antibiotics in the initial management of ABM. These findings are consistent with reports coming from Taiwan recently [9, 18, 19]. Fortunately, so far, we have not encountered vancomycin-resistant coagulase-negative staphylococci strains or colistin-resistant* Acinetobacter* strains.

Finally, in an attempt to identify independent predictors of mortality in patients with ABM, many studies had been conducted. The concluded prognostic factors among these studies were diverse [9, 10, 18, 19, 22, 23]. Our study revealed many probable prognostic factors; however, only the presence of underlying diseases, hypotension, and inappropriate treatment were found to be independent predictors of mortality by multivariate logistic regression analysis.

This hospital-based study has the following limitations. First, the study was retrospective rather than prospective, and this design did not allow us to obtain additional details such as severity of the disease and long-term follow-up to evaluate the long-term sequelae of meningitis in our patients. Second, it was performed at a single hospital; the results may not be applicable to other hospitals. Third, we included patients who had a positive CSF culture or positive CSF bacterial antigen test.

Despite these limitations, we believe that our study remains the largest to date to provide comprehensive information on the epidemiology of ABM in Qatar.

In conclusion, our study revealed that there is a change in the predominantly affected age group and common causative agents of ABM. Coagulase-negative staphylococci species are the common causative agent in Qatar with majority of infections occurring nosocomially. More than 90% of all implicated coagulase-negative staphylococci strains were oxacillin-resistant. Thus, improving our infection control programs in addition to enhancing antimicrobial stewardship is essential to overcome this problem.

## Figures and Tables

**Table 1:** Demographic and clinical data of the 117 patients involved in this study.

Variable	Number (%), mean ± SD (range)
*Gender*	
M	81 (69.2)
F	29 (30.8)
*Age* (mean ± SD), years	26.4 ± 22.3 (2–74)
*Age group (years)*	
<1	26 (22.2)
1–5	11 (9.4)
6–14	6 (5.1)
15–24	12 (10.3)
25–34	13 (11.1)
35–44	17 (14.5)
45–54	23 (19.7)
55–64	6 (5.1)
≥65	3 (2.6)
*Nationality*	
Qatari	28 (23.9)
Non-Qatari	82 (76.1)
*Underlying conditions*	
Diabetes mellitus	9 (7.7)
Hypertension	26 (22.2)
Head injury	6 (5.1)
Neurosurgery	54 (46.2)
Alcoholic	4 (3.4)
Prematurity	7 (6.0)
Liver cirrhosis	1 (0.9)
Otitis media	5 (4.3)
Malignancy	8 (6.8)
Immunosuppression	2 (1.7)
*Clinical presentation*	
Fever	110 (94)
Mental alteration	55 (47)
Headache	43 (36.8)
Vomiting	35 (29.9)
Meningism	31 (26.5)
Seizures	23 (19.7)
Bulging fontanel	16 (13.7)
Hypotension (BP < 90/60 mmHg)	12 (10.2)
Focal signs	11 (9.4)
Photophobia	7 (6.0)
Behavioral changes	3 (2.6)
Petechial rash	3 (2.6)
*Complications*	
Hydrocephalus	19 (16.2)
Ischemic stroke	5 (4.3)
Brain abscess	4 (3.4)
Subdural empyema	1 (0.9)
Adrenal insufficiency	1 (0.9)
Vasculitis	1 (0.9)

**Table 2:** Clinical data of the 117 patients involved in this study.

Variable	Number (%), mean ± SD (range)
*Acquisition of infection*	
Community-acquired	59 (50.4)
Nosocomial	58 (49.6)
*CSF*	
Cells/*µ*L	3880.4 ± 8654.6 (20–66000)
Neutrophils%	74.1 ± 26.1 (1–99)
Lymphocytes%	22.9 ± 24.8 (1–98)
Protein (g/dL)	222.8 ± 205.9 (38–936)
Glucose (mmol/L)	2.1 ± 1.6 (0.1–6)
Positive Gram stain	93 (79.5)
Positive culture	112 (95.7)
Positive latex agglutination	23 (19.7)
*Type of microorganism*	
Gram-positive	62 (53)
Gram-negative	55 (47)
*Positive blood culture*	37 (31.6)
*Antimicrobial therapy*	
Appropriate	97 (82.9)
Inappropriate	20 (17.1)
*Outcome *	
Died	14 (12.0)
Alive	103 (88.0)

**Table 3:** Distribution of different isolates in relation to setting of acquisition of meningitis.

Microorganism	Setting of acquisition		Total
	Nosocomial *N* (%)	Community-acquired *N* (%)	
Gram-positive			
*Abiotrophia* species	1 (100)	0	1
*Enterococcus faecalis *	5 (71.4)	2 (28.6)	7
*Enterococcus gallinarum *	1 (100)	0	1
*Gemella haemolysans*	1 (100)	0	1
*Leuconostoc* species	1 (100)	0	1
*Listeria monocytogenes*	0	3 (100)	3
*Staphylococcus aureus*	0	1 (100)	1
*Staphylococcus capitis*	2 (100)	0	2
*Staphylococcus epidermidis*	14 (87.5)	2 (12.5)	16
*Staphylococcus haemolyticus*	2 (100)	0	2
*Streptococcus agalactiae*	0	3 (100)	3
*Streptococcus bovis II*	0	1 (100)	1
*Streptococcus intermedius *	0	1 (100)	1
*Streptococcus milleri *	0	1 (100)	1
*Streptococcus mitis *	0	1 (100)	1
*Streptococcus pneumoniae *	0	19 (100)	19
*Streptococcus salivarius*	0	1 (100)	1
Gram-negative			
*Acinetobacter baumannii*	8 (100)	0	8
*Acinetobacter lwoffii *	2 (66.7)	1 (33,3)	3
*Brucella *spp.	0	1 (100)	1
*Chryseobacterium* (*Flavobacterium*) *meningosepticum*	0	1 (100)	1
*Chryseobacterium indologenes*	0	1 (100)	1
*Enterobacter aerogenes*	1 (100)	0	1
*Enterobacter cloacae*	3 (100)	0	3
*Escherichia coli*	3 (75)	1 (25)	4
*Haemophilus influenzae*	1 (50)	1 (50)	2
*Klebsiella pneumoniae *ssp.* pneumoniae*	7 (58.3)	5 (41.7)	12
*Neisseria meningitides*	0	11 (100)	11
*Pseudomonas aeruginosa*	5 (100)	0	5
*Pseudomonas putida*	1 (100)	0	1
*Salmonella *group B	0	1 (100)	1
*Serratia marcescens *	0	1 (100)	1
Total	**58**	**59**	**117**

**Table 4:** Distribution of microorganisms in relation to the age group.

Microorganism	Age group									Total
	<1	1–5	6–14	15–24	25–34	35–44	45–54	55–64	≥65	
*Abiotrophia *species	0	0	0	0	0	1	0	0	0	1 (0.8)
*Acinetobacter baumannii*	0	0	0	0	0	2	3	2	1	8 (6.8)
*Acinetobacter lwoffii*	0	1	1	0	1	0	0	0	0	3 (2.6)
*Brucella *spp.	0	0	0	0	0	0	1	0	0	1 (0.8)
*Chryseobacterium (Flavobacterium) meningosepticum*	1	0	0	0	0	0	0	0	0	1 (0.8)
*Chryseobacterium indologenes*	1	0	0	0	0	0	0	0	0	1 (0.8)
*Enterobacter aerogenes*	1	0	0	0	0	0	0	0	0	1 (0.8)
*Enterobacter cloacae*	0	0	0	0	0	1	2	0	0	3 (2.6)
*Enterococcus faecalis*	2	1	0	0	0	1	2	1	0	7 (5.9)
*Enterococcus gallinarum*	0	0	0	0	0	0	1	0	0	1 (0.8)
*Escherichia coli*	2	1	0	0	0	1	0	0	0	4 (3.4)
*Gemella haemolysans*	0	0	0	1	0	0	0	0	0	1 (0.8)
*Haemophilus influenzae*	0	1	0	1	0	0	0	0	0	2 (1.6)
*Klebsiella pneumoniae *ssp.* pneumoniae*	2	0	0	2	1	1	4	2	0	12 (10.3)
*Leuconostoc *species	0	0	0	0	0	0	1	0	0	1 (0.8)
*Listeria monocytogenes*	3	0	0	0	0	0	0	0	0	3 (2.6)
*Neisseria meningitidis*	0	1	0	3	3	1	2	0	1	11 (9.4)
*Pseudomonas aeruginosa* (PSA)	0	0	1	0	2	1	1	0	0	5 (4.2)
*Pseudomonas putida*	0	0	0	0	1	0	0	0	0	1 (0.8)
*Salmonella *group B	1	0	0	0	0	0	0	0	0	1 (0.8)
*Serratia marcescens*	1	0	0	0	0	0	0	0	0	1 (0.8)
*Staphylococcus aureus*	0	0	1	0	0	0	0	0	0	1 (0.8)
*Staphylococcus capitis*	0	0	0	0	0	1	1	0	0	2 (1.6)
*Staphylococcus epidermidis*	6	2	0	2	1	3	1	1	0	16 (13.6)
*Staphylococcus haemolyticus*	0	0	0	0	1	1	0	0	0	2 (1.6)
*Streptococcus agalactiae*	2	0	0	0	0	0	0	0	1	3 (2.6)
*Streptococcus bovis II*	1	0	0	0	0	0	0	0	0	1 (0.8)
*Streptococcus intermedius*	0	0	0	0	0	1	0	0	0	1 (0.8)
*Streptococcus milleri*	0	0	0	1	0	0	0	0	0	1 (0.8)
*Streptococcus mitis*	0	1	0	0	0	0	0	0	0	1 (0.8)
*Streptococcus pneumoniae*	3	2	3	2	3	2	4	0	0	19 (16.2)
*Streptococcus salivarius*	0	1	0	0	0	0	0	0	0	1 (0.8)

Total	26 (22.2)	11 (9.4)	6 (5.1)	12 (10.3)	13 (11.1)	17 (14.5)	23 (19.7)	6 (5.1)	3 (2.6)	117 (100)

**Table 5:** Antimicrobial resistance rate of Gram-positive CSF isolates.

Microorganisms	TNP	pen	amp	oxc	eryt	clind	amclv	cotr	cfr	van	line	teic
*Abiotrophia *spp.	1	0	0	NT	0	0	0	0	NT	0	NT	NT
*Enterococcus faecalis*	7	NT	0	NT	NT	NT	NT	NT	NT	0	0	0
*Enterococcus gallinarum*	1	NT	0	NT	NT	NT	NT	NT	NT	1 (100)	0	0
*Gemella haemolysans*	1	0	0	NT	NT	NT	NT	NT	0	0	NT	NT
*Leuconostoc *species	1	0	0	NT	NT	0	0	NT	1 (100)	1 (100)	0	0
*Listeria monocytogenes*	3	0	0	NT	NT	NT	NT	0	NT	NT	NT	NT
*Staphylococcus aureus*	1	1 (100)	1 (100)	NT	0	0	0	0	NT	0	0	0
Coagulase-negative staphylococci	20	20 (100)	20 (100)	19 (95)	19 (95)	10 (50)	20 (100)	9 (45)	NT	0	0	0
*Streptococcus *spp.	8	1 (0.2)	1 (0.2)	0	1 (0.2)	1 (0.2)	0	1 (0.2)	1 (0.2)	0	0	0
*Streptococcus pneumoniae*	19	6 (31.5)	6 (31.5)	6 (31.5)	6 (31.5)	6 (31.5)	6 (31.5)	3 (15.7)	3 (15.7)	0	0	0

TNP: total number of patients; pen: penicillin; amp: ampicillin; oxc: oxacillin; eryt: erythromycin; clind: clindamycin; amclv: amoxicillin/clavulanic acid; cotr: cotrimoxazole; cfr: ceftriaxone; van: vancomycin; line: linezolid; teic: teicoplanin; NT: not tested.

**Table 6:** Antimicrobial resistance rate of Gram-negative cerebrospinal fluid isolates.

Microorganisms	TNP	cfr	gen	fep	taz	cip	amclv	ctz	amk	mem	pen	rif	col	tig
*Acinetobacter *spp.	11	8 (72.7)	8 (72.7)	8 (72.7)	8 (72.7)	7 (63.6)	NT	7 (63.6)	7 (63.6)	7 (63.6)	NT	NT	0	0
*Brucella *species	1	NT	NT	NT	NT	NT	NT	NT	NT	NT	NT	NT	NT	NT
*Chryseobacterium *spp.	2	1 (50)	2 (100)	0	1 (50)	0	NT	2 (100)	2 (100)	2 (100)	NT	NT	2 (100)	2 (100)
*Enterobacter *species	4	3 (75)	0	1 (25)	3 (75)	0	4 (100)	3 (75)	0	0	NT	NT	NT	NT
*Escherichia coli*	4	1 (25)	1 (25)	1 (25)	0	1 (25)	1 (25)	1 (25)	0	0	NT	NT	NT	NT
*Haemophilus influenzae*	2	0	NT	0	0	NT	0	NT	NT	NT	NT	NT	NT	NT
*Klebsiella pneumoniae *ssp.* pneumoniae*	12	3 (25)	0	3 (25)	2 (16.6)	2 (16.6)	7 (58.3)	4 (33.3)	0	0	NT	NT	0	0
*Neisseria meningitidis*	8	0	NT	0	0	2 (25)	0	NT	NT	0	0	2 (25)	NT	NT
*Pseudomonas aeruginosa *	5	NT	0	0	0	0	NT	0	0	0	NT	NT	0	0
*Pseudomonas putida*	1	NT	0	0	0	0	NT	0	0	0	NT	NT	0	0
*Salmonella *group B	1	0	1 (100)	0	0	0	0	0	0	0	NT	NT	NT	NT
*Serratia marcescens*	1	0	0	0	0	0	1 (100)	0	0	0	NT	NT	NT	NT

TNP: total number of patients; cfr: ceftriaxone; gen: gentamicin; fep: cefepime; taz: piperacillin/tazobactam; cip: ciprofloxacin; amclv: amoxicillin/clavulanic acid; ctz: ceftazidime; amk: amikacin; mem: meropenem; pen: penicillin; rif: rifampicin; col: colistin; tig: tigecycline; NT: not tested.

**Table 7:** Results of univariate analysis of in-hospital mortality predictors.

Variable	Unadjusted odds ratio (95% CI)	*P* value
Presence of underlying diseases	2.4 (1.4–3.9)	0.001
Nosocomial infection	3.2 (1.5–6.5)	0.1
Multidrug-resistant episodes	4.7 (3.8–5.7)	0.08
Mental alteration	4.9 (1.0–24.0)	0.06
Hypotension	2.3 (0.7–7.3)	0.003
Inappropriate treatments	1.9 (1.2–3.07)	0.01

**Table 8:** Results of multivariate analysis of in-hospital mortality independent factors.

Variable	Adjusted odds ratio (95% CI)	*P* value
Presence of underlying diseases	1.8 (1.03–3.2)	0.02
Hypotension	3.2 (1.4–7.3)	0.04
Inappropriate treatments	1.7 (1.0–2.8)	0.01
